# Perioperative Hypothermia in Children

**DOI:** 10.3390/ijerph18147541

**Published:** 2021-07-15

**Authors:** Marcus Nemeth, Clemens Miller, Anselm Bräuer

**Affiliations:** Department of Anaesthesiology, University Medical Centre Goettingen, 37075 Goettingen, Germany; marcus.nemeth@med.uni-goettingen.de (M.N.); anselm.braeuer@med.uni-goettingen.de (A.B.)

**Keywords:** perioperative, hypothermia, children, newborn, infant, paediatric, anaesthesiology, warming strategy, risk

## Abstract

Background: First described by paediatric anaesthesiologists, perioperative hypothermia is one of the earliest reported side effects of general anaesthesia. Deviations from normothermia are associated with numerous complications and adverse outcomes, with infants and small children at the highest risk. Nowadays, maintenance of normothermia is an important quality metric in paediatric anaesthesia. Methods: This review is based on our collection of publications regarding perioperative hypothermia and was supplemented with pertinent publications from a MEDLINE literature search. Results: We provide an overview on perioperative hypothermia in the paediatric patient, including definition, history, incidence, development, monitoring, risk factors, and adverse events, and provide management recommendations for its prevention. We also summarize the side effects and complications of perioperative temperature management. Conclusions: Perioperative hypothermia is still common in paediatric patients and may be attributed to their vulnerable physiology, but also may result from insufficient perioperative warming. An effective perioperative warming strategy incorporates the maintenance of normothermia during transportation, active warming before induction of anaesthesia, active warming during anaesthesia and surgery, and accurate measurement of core temperature. Perioperative temperature management must also prevent hyperthermia in children.

## 1. Definition

The normal temperature measured rectally in children up to five years of age is 36.5–38.0 °C [1]. Hypothermia is defined as a core temperature below 36.5 °C or 36.0 °C in children up to five years of age and children older than five years, respectively. A core temperature below 35.0 °C can be defined as severe perioperative hypothermia.

## 2. History

The first description of perioperative hypothermia dates from 1847, making it one of the earliest reported side effects of general anaesthesia [2]. In the 1950s and 1960s, hypothermia was associated with adverse outcomes, especially in paediatric anaesthesia. Hypothermic infants were lethargic and had respiratory depression [3]; severe perioperative hypothermia was associated with mortality [4]. Therefore, the first intraoperative warming devices were used in paediatric patients. In 1953, an electrical heating blanket was described [5], followed by the first use of a circulating water mattress for infants (1962) [6] and the development of the first forced-air warming system for infants (1973) [7].

## 3. Incidence

Although paediatric anaesthesiologists were the first to observe, describe, and implement effective measures against perioperative hypothermia, children still often become hypothermic. This may be due to the vulnerable physiology of this cohort, but also due to insufficient perioperative warming.

Unfortunately, most of the studies in children report only the incidence of postoperative hypothermia, while intraoperative hypothermia is more frequent. In the largest retrospective study with 6737 children by Görges et al. [8], hypothermia was observed in 45% of patients. Pearce et al. reported a 52% incidence of hypothermia in 717 children [9]. Core temperature was measured in only 74% of the patients, and only about 50% received active warming with forced-air. Conflicting results were reported in several other studies in neonates and infants. In a retrospective study on premature infants undergoing laparotomies for necrotizing enterocolitis by Sim et al., perioperative hypothermia was seen in 85% [10]. Similarly, in a retrospective study by Cui et al., the hypothermia rate in neonates was 82% [11]. In a small study by Ongun et al. with infants undergoing craniosynostosis repair, the hypothermia rate was 83% [12]. However, in a large study by Thompson et al. that evaluated 933 infants undergoing open or endoscopic craniectomy for craniosynostosis repair, the rate of severe intraoperative hypothermia was 22% and 26%, respectively [13].

On the other hand, some studies demonstrate that with an adequate protocol, hypothermia rates can be below 10% [14,15,16,17], even in preterm infants [18]. Thus, it seems that the rate of perioperative hypothermia depends more on the actual warming strategy, and less on patient factors, regardless of age or surgical procedure.

## 4. Development

Four aspects contribute to the proneness of children, and particularly neonates, to greater heat loss: (i) their regulatory capacity is less effective than in adults, (ii) their reduced weight-to-surface-area (WSA) ratio, (iii) their increased heat loss from the head, and (iv) their limited stores of subcutaneous fat for thermal insulation [19]. Further, neonates are not able to move to warmer places or put on more clothes on their own to make use of behavioural regulation, the most powerful thermoregulatory effect [20].

Warm-blooded animals rely on a functioning system for homeostatic regulation of body temperature to maintain their thermo-neutral range. This is the individual range in which the body does not react autonomously to the environmental temperature through sweating or thermoregulatory vasoconstriction. To equal heat loss during anaesthesia, heat generation is activated if the autonomic thermoregulatory thresholds are exceeded. When the human body temperature is too low, the primary autonomic defences are shivering and arteriovenous shunt vasoconstriction in the body’s periphery [21].

As the effector mechanisms of skeletal muscle stimulation are minimal [22], heat generation by shivering is limited in neonates. They depend on non-shivering thermogenesis [20]. Non-shivering thermogenesis initiates a neuroendocrine pathway triggered by increased sympathetic activity, which leads to the release of thyroid-stimulating hormone and subsequently an increase in triiodothyronine (T_3_) through the conversion of thyroxin (T_4_), as well as a release of norepinephrine in the brown adipose tissue [22]. These pathways upregulate the thermogenin protein in the brown adipose tissue, thereby uncoupling mitochondrial oxidative phosphorylation leading to heat production [22].

The brown adipose tissue is most prominent in the interscapular area [23], but contrary to popular belief still has some activity in the later age [24] and attracts growing interest for obesity and diabetes research [25]. However, it is the main thermogenic organ in neonates, and can double heat production in infants [26]. Thermogenin exhibits a major increase around the 32nd week of gestation [27]. In extremely low birth weight (ELBW) neonates (<1000 g), inefficient thermoregulation is associated with lower thermogenin levels [22,27]. Sick neonates can also be deficient in thermogenin.

Further, ELBW neonates have poor vasomotor control at birth [28] and are unable to exhibit peripheral vasoconstriction to preserve heat [22]. Nevertheless, in full-term newborns, thermoregulation is generally well-developed at birth [21]. In infants, thermoregulatory response thresholds are well-preserved [29], and no worse than in adults [20]. Plattner et al. did not find any evidence of non-shivering thermogenesis at core temperatures 2 °C below the vasoconstriction threshold during anaesthesia [30]. It appears that infants, like adults, are unable to increase their metabolic rate in response to intraoperative hypothermia [30].

This shifts the focus of perioperative temperature management more towards the small thermal mass and reduced WSA ratio of children, which explains the greater susceptibility to environmental disturbances than adults [21]. Differences in non-evaporative heat losses per °C in temperature between the skin and the room, and per body surface area are the same as with exposed skin, as in adults. However, in children, a high room temperature is much more important, due to the reduced WSA ratio. This fact renders children to become hypothermic much faster in a cold environment [31]. The area of the operating room (OR) is usually surrounded by cold air in the corridors, resulting in a larger temperature gradient between the patient and the environment. The heat loss along this gradient can be crucial, particularly by radiation and convection [21]. Radiative heat loss, especially due to the large head, is the most important route of heat transfer after the first postnatal week [32]. Convective heat loss is typically predominant when an infant is carried through the cool air to the OR. Commonly administered benzodiazepines for premedication can lead to a dose-dependent drop in core temperature [33], although data for children are lacking.

All these preconditions aggravate the risk of perioperative hypothermia. When anaesthesia is induced, patients need to rely on their autonomic defences and external thermal management [20], even during the initial phase of redistribution. The higher mass in the torso [34] and the relatively low mass in the extremities compared to adults could weaken the drop in core temperature [35] (p. 55), but this does not compensate for the heat loss that is caused by the mechanisms described above. However, the drop in core temperature from baseline is lower in infants than in older children [36].

Any type of anaesthesia can affect thermal homeostasis. Drugs for general anaesthesia produce a decrease in vasoconstriction and shivering thresholds [21]. Intravenous (IV) anaesthetics affect thermal homeostasis in a concentration-dependent manner and inhalational anaesthetics affect thermal homeostasis in a non-linear, concentration-dependent manner (the higher the concentration, the disproportionally more the decrease). Volatile anaesthetics were also shown to inhibit the non-shivering thermogenesis [37], which is not clinically relevant [30]. The iatrogenic vasodilatation restricts the thermal response of vasoconstriction in the body’s periphery, leading to a redistribution of heat from the core to the periphery. Caudal anaesthesia has little effect on the thermoregulatory threshold for vasoconstriction [29].

During surgery, the development of perioperative hypothermia in children is enhanced through the administration of cold fluids, the application of dry anaesthetic gases, and wound exposure.

## 5. Risk Factors

In children, a higher risk of perioperative hypothermia is correlated with low body weight and a more immature thermoregulatory system [38,39]. The heat loss of a naked newborn at birth in an environmental temperature of 23 °C corresponds with the heat loss of an unclothed adult at 0 °C [40]. Thus, it is of major importance to maintain their thermo-neutral range. Small and sick children have a narrow thermo-neutral range, and are prone to thermal instability [40]. Several external factors were identified to be associated with perioperative hypothermia, for example, invasive procedures and inadequate core temperature monitoring.

Tander et al. identified risk factors for perioperative hypothermia in 60 neonates, and found major intestinal surgery and an OR temperature less than 23 °C to be the most important [38]. In a prospective case–control study including 108 infants treated in the neonatal intensive care unit (NICU), Morehouse et al. demonstrated that hypothermia was most frequent in infants when surgery was performed in the OR compared to the NICU [39]. The incidence of hypothermia was nearly five times higher in the OR group (65.5% and 13.2%, respectively). Furthermore, neonates receiving interventional cardiac procedures are known to be at significant risk of hypothermia [41,42].

In studies that investigated a broader age range, hypothermia was more frequent in older children compared to younger ones, and associated with type and duration of surgery (e.g., major orthopaedic surgery), low baseline temperature, high blood loss, and transfusion requirement [9,16,36]. This is particularly interesting as, adequate warming measures, older age, and a long duration of anaesthesia are considered protective factors against postoperative hypothermia [43].

## 6. Adverse Events

Hypothermia in the paediatric patient can lead to adverse events which can range from thermal discomfort to increased morbidity and mortality [44]. Though robust data are lacking, one can assume that children may also be affected by the detrimental adverse events of hypothermia, similar to adults [45,46]. This may be true when considering: (i) pharmacokinetics and pharmacodynamics (essentially muscle relaxants), (ii) platelet function, coagulation, and blood loss, (iii) cardiocirculatory and respiratory complications, (iv) wound healing and surgical site infections (SSI), and (v) thermal discomfort [9,35,47]. On the other hand, in children, there is no clarity about the short- and long-term consequences of intraoperative hypothermia and whether hypothermia may not be even protective under certain circumstances [10]. However, except for mild therapeutic hypothermia in neonates with moderate to severe perinatal asphyxia [48], there is currently no evidence that hypothermia improves outcomes in any condition such as cardiac arrest [49,50], extracorporeal membrane oxygenation [51], and traumatic brain injury [52].

### 6.1. Neonates and Preterm Infants

**Figure 1:**
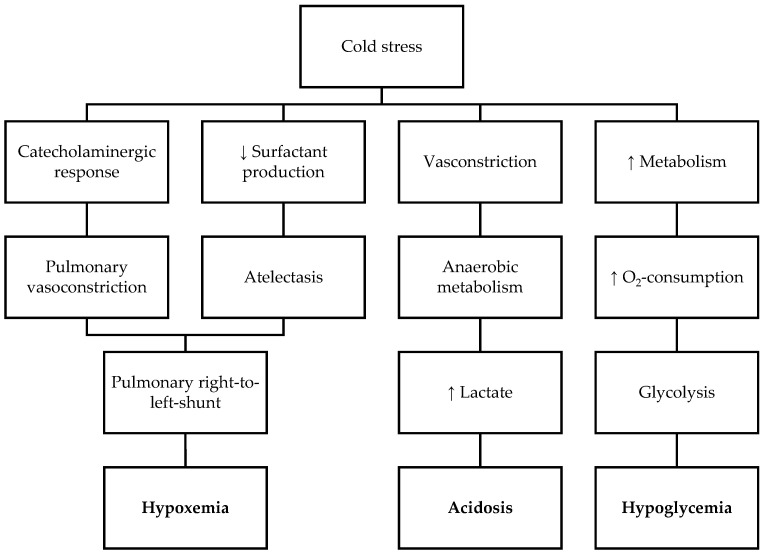
Pathophysiological pathways resulting from adverse events induced by cold stress in neonates.

Cold stress may induce multiple physiological pathways such as catecholaminergic response, vasoconstriction, increased metabolism, and decreased surfactant synthesis (Figure 1). This may lead to pulmonary hypertension, tissue hypoxia, arterial hypotension and hypoperfusion of vital organs, metabolic acidosis, and hypoglycaemia [14,41,44]. The possible consequences may be apnoea and the need for mechanical ventilation, arrhythmias, increased risk of infections, prolonged length of hospital stay, poor neurologic outcome, and even death [14,38,53].

In very low birth weight (VLBW) infants (<1500 g), transient neonatal hypothermia after birth is associated with intraventricular haemorrhage and death [54]. Perioperatively, studies report different observations, and it is unclear under which circumstances and how often detrimental effects occur in the phase after birth. In a study by Morehouse et al. neonates suffering from perioperative hypothermia had significantly more respiratory adverse events, required six times more thermoregulatory interventions, were five times more likely to receive cardio-circulatory support, and were three times more likely to receive respiratory interventions [39]. On the other hand, Sim et al. found only a higher transfusion requirement in their retrospective analysis of 49 preterm infants with necrotizing enterocolitis requiring laparotomy [10].

### 6.2. Infants and Children

In a retrospective study by Görges et al., hypothermia was associated with wound disruption, whereas SSI and transfusion requirement were not [8]. Pearce et al. showed an association of hypothermia with higher blood loss and a need for blood transfusions [9]. In children with a median age of 15 years that underwent spinal surgery, another retrospective study by Görges et al. reported that prewarming was associated with higher core temperatures and lower consumption of packed red blood cell transfusion [55].

Thus, some specific adverse events are associated with perioperative hypothermia in the paediatric population. This is true for increased blood loss, transfusion [9,55], and impaired wound healing [8]. Reports about SSI are conflicting [56,57].

## 7. Monitoring of Core Temperature

As body temperature is one of the classic vital signs, its perioperative monitoring is a key responsibility of anaesthesiologists [58]. Any perturbation of body temperature must be detected. This applies not only to the prevention of hypothermia, but also to the detection of any state of hyperthermia, be it malignant, iatrogenic, drug-induced, or fever, which requires immediate interventions. An accurate measuring method is therefore indispensable for proper temperature management.

Given that the extremities are usually 2 to 4 °C cooler than the core in the hospital environment [59], and the skin surface is yet cooler [60], peripheral measurement is inaccurate in the perioperative setting. The gold standard that best reflects body temperature is the core temperature, as it is the dominant input to autonomic thermoregulatory control [58]. It can be measured in well-perfused sites with blood from the core.

### 7.1. Core Sites

**Figure 2:**
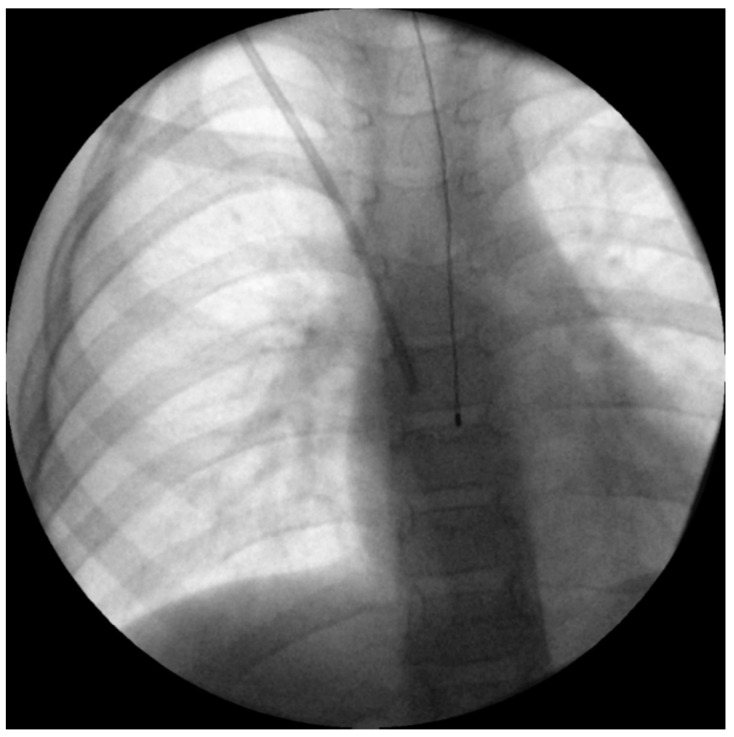
Intraoperative X-ray of a 6-year-old boy undergoing Broviac catheter implantation with appropriate depth of oesophageal temperature probe inserted, according to the estimation method by Whitby et al. [64].

Although the best single estimate of core temperature is considered the pulmonary artery [58], its approach is completely invasive, and hence not suitable for children undergoing non-cardiac surgery. A more commonly used alternative is an oesophageal temperature probe [61,62]. Inserting the probe deep enough to reach the lower third of the oesophagus is important to accurately estimate core temperature [63]. Correctly placed, it lies directly between the left atrium and the descending aorta, and is therefore far away from the potentially cooling airway (Figure 2). Insertion depth formulas for children such as those of Whitby et al. [64] or Hong et al. [65] can help estimate the appropriate insertion depth (Table 1).

When oesophageal monitoring is not feasible, e.g., due to surgical procedure, nasopharyngeal temperature is an adequate alternative and a reliable surrogate for core temperature [58,66]. While nasopharyngeal probes should be inserted 10–20 cm in adults [67], insertion depth in children is unclear. The accuracy of nasopharyngeal probes is only minimally influenced by uncuffed endotracheal tubes [68]. However, both methods are still invasive, and can cause harm to the vulnerable mucosa, although the incidence of such events in children is unknown.

In theory, the tympanic membrane is also a true core site, but practically is difficult to reach as the aural canal is not straight. Clinicians often do not insert the probes far enough to reach the membrane itself, and measure the skin temperature of the canal instead [58]. Additionally, infrared aural canal systems are too imprecise for perioperative use [69]. Nevertheless, current data in children is lacking.

### 7.2. Other Sites

Axillary temperature, which is commonly used in NICUs [14] and in PACUs [70] is close to core temperature, at least in normal temperature ranges, but not as accurate. This is also true for oral probes [58]. In awake children, these methods can be applied reasonably. Other near-core sites such as rectal or bladder have a lag of temperature changes in adults, but probably less so in children [58]. The better-tolerated temporal artery thermometers have only limited sensitivity [71].

### 7.3. Recommendations

German guidelines on prevention of perioperative hypothermia recommend rectal temperature measurement pre- and postoperatively [1]. During anaesthesia, oesophageal or nasopharyngeal probes can be used [1].

For perioperative temperature monitoring, we suggest the measurement methods listed in Table 2.

### 7.4. Perspectives

Ideally, a non-invasive, quick to apply measurement system would be preferable, which could be used in the entire perioperative period [17]. Presently, several non-invasive thermometers are available, and are gaining more and more interest in children’s medicine [17,72,73]. However, to date, neither technique has been evaluated thoroughly in children [74]. The ideal non-invasive thermometer would be very precise, regardless of age, temperature range, and duration of measurement.

## 8. Prevention

### 8.1. Warming Therapy during Transport

It is crucial to have a thermal management strategy before a child is brought to the OR. If the child is already hypothermic upon arrival, even the best warming strategy is inefficient. Thus, it is inadequate to start warming therapy in these extremely vulnerable patients after induction of anaesthesia. Convective heat loss at transportation through cold surroundings, such as corridors from the ward, must therefore be avoided. All children should be dressed for as long as possible and covered with blankets when in an ideally prewarmed bed to prevent cooling of the body’s periphery.

Further, preterm infants and neonates should be transported in a warmed incubator. In high-risk patients (e.g., ELBW neonates) it should be evaluated whether surgery in the NICU is possible [39]. If transport is required, then there are several measures which can contribute to minimizing heat losses. The transport device must be appropriate, ideally prewarmed, and provided with a radiant heater or warming mattress. The system should be covered with a cap during transport. It is recommended to keep the incubator plugged in during the surgical procedure [39]. Optionally, a warming pad could be used during transport [16]. Checklists for the preparation of transport have been shown to be effective [16,41].

### 8.2. Warming before Induction of Anaesthesia

The OR must be warmed to a temperature around the thermo-neutral range that allows the children to remain warm before induction of anaesthesia. It must be considered that this can take up to 60 min [75]. In general, a room temperature of 32 °C is adequate for neonates and room temperatures between 24 °C and 30 °C are adequate for infants. Warm OR temperatures are effective to attenuate the initial drop in core temperature after induction of anaesthesia [75] and to prevent hypothermia [38].

**Figure 3:**
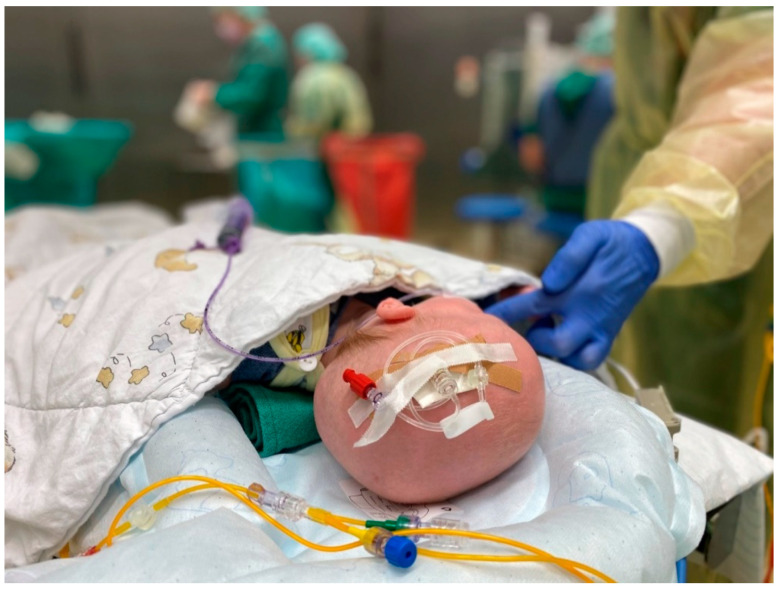
Use of an underbody, forced-air, warming blanket and insulation cover to efficiently prewarm a 4-month-old child.

In addition to the elevated room temperature, it is of utmost importance to start active warming therapy before induction of anaesthesia. The most convenient way is to place the child on an already warmed underbody, forced-air, warming blanket. Then, the child is undressed to establish standard monitoring and the uncovered parts of the body are insulated. In this way, heat losses can be reduced as much as possible (Figure 3). Insulation can reduce heat losses from the covered skin by up to 68 % when a thick hospital duvet is used [76].

Active prewarming is recommended in adult patients by several guidelines [1,77] to reduce the redistribution of heat from the warm core of the body to the colder periphery [78]. Although this effect in small children is less pronounced, small children depend on a higher surrounding temperature to prevent cooling when they are undressed. From a physiologic point of view, the rationale pertains more to active warming therapy to prevent cooling before induction of anaesthesia, rather than active prewarming to reduce redistribution of heat. Still, from the practical point of view, it is the same measure. In adults, active prewarming is recommended to last more than 10 min [79] but there are no data about children. As the periphery of small children has less mass and more surface area for warming therapy, prewarming will probably need less time. Following these principles, it is very unlikely for a small child to become hypothermic before a standard procedure.

**Figure 4:**
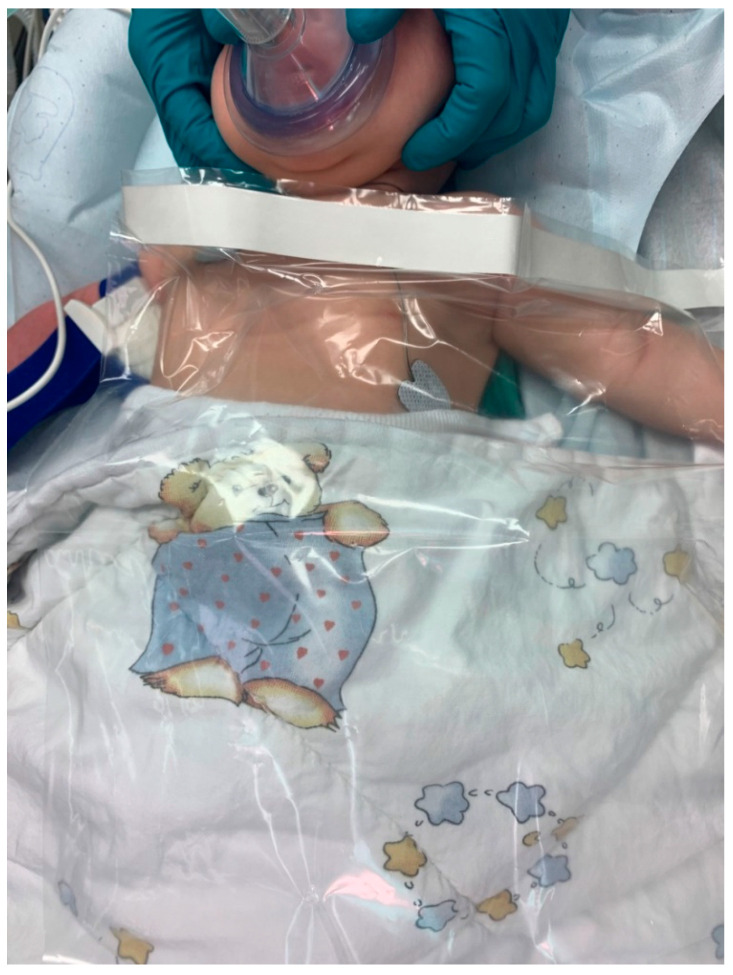
Continued warming therapy after mask-induction during IV-cannulation of an infant.

However, if preparation is protracted (e.g., when extended surgery requires placement of arterial and central venous lines), it is often impossible to insulate large parts of the body surface, leading to hypothermia. This is especially true for small infants [80]. However, use of covers and insulation should be continued as long as possible in such circumstances (Figure 4).

### 8.3. Warming Therapy during Anaesthesia

During anaesthesia and surgery, active warming therapy must be continued. The reduced WSA ratio render children to become hypothermic much faster in a cold environment; on the other hand, this reduced WSA ratio allows for faster (re-)warming than adults by using a forced-air warmer [31]. Thus, it is usually easy to keep paediatric patients normothermic during surgery [81,82]. Conductive warmers are rarely used in infants for this purpose, mainly as they are not as effective as forced-air warmers [83].

**Figure 5:**
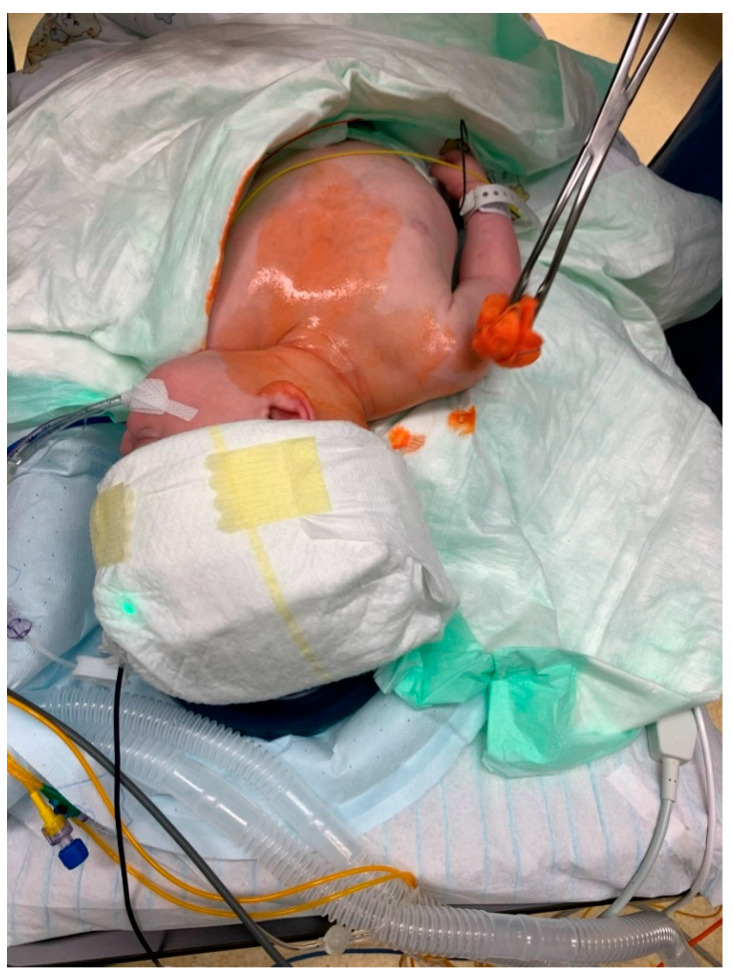
Insulation of the body parts (e.g., napkin for the head) that cannot be actively warmed to reduce heat losses during washing and draping of an infant.

It has been shown in adults that pausing forced-air warming during washing and draping can increase the risk of intraoperative hypothermia in a time-dependent manner [84]. This effect can be expected to be even more important in paediatric patients. Therefore, paediatric patients should be continuously warmed, if possible. To date, there is no evidence that warming during washing and draping increases the risk of infection [85]. The parts of the body that cannot be warmed actively should be insulated to reduce heat loss (Figure 5).

Intraoperatively, active warming must be continued, and irrigation solutions should be warmed to body temperature. Heat and moisture exchangers reduce the evaporative heat loss from the airways. This is more important in small children than in adults, as children have higher minute ventilation per kilogram of body weight [86].

### 8.4. Infusion Warming

In children, an intraoperative maintenance infusion of 10 mL/kg/h is commonly used. The infusion rate is even higher when additional requirements are considered (e.g., fluid or blood losses) [87]. This would correspond to an infusion rate of about 700 mL/h in an adult weighing 70 kg, and several guidelines for the prevention of perioperative hypothermia [1,77] recommend infusion warming in adults for high infusion rates of >500 mL/h. However, many paediatric anaesthesiologists do not use infusion warming for the background infusion and do not have high hypothermia rates [17,81,82,88,89]. The continuous heat losses from the maintenance infusion can usually be offset by forced-air warming. However, this situation changes when massive fluid administration and blood products must be used. Then, it is particularly important to warm fluids and blood products with a blood and fluid warmer [90]. In general, the heat loss by unwarmed infusion depends mainly on the volume and the temperature of the fluid at the distal end of the infusion tubing. Therefore, the infusion warming device used should provide a high fluid temperature at the IV cannula. In paediatric patients, flow rates are relatively small compared to adults. Therefore, special attention should be paid to the length of the infusion tubing after the heat exchanger, as relevant heat losses of the infusion during transit from the heat exchanger to the IV cannula will occur [91,92,93]. Alternatively, the infusion tubing after the heat exchanger can be placed under the forced-air warming blanket to prevent the cooling of the fluid after transit from the heat exchanger. Infusion warming devices that release aluminium into the infusion fluid should not be used [94].

### 8.5. Warming Therapy after Anaesthesia

Similar to the management of adults, extubation should not be performed when children are hypothermic. Safe temperature limits at which anaesthesia can be discontinued are unknown [1]. If hypothermic, children should be rewarmed during anaesthesia and then extubated.

Every child should return to a prewarmed bed after anaesthesia. In the postoperative phase, core temperature should be measured regularly to detect hypothermia developed after continuous measurement ceases (e.g., through transport to postoperative care unit). For this purpose, a non-invasive method should be used if possible [1]. In patients at risk of postoperative hypothermia, monitoring of core temperature should be continued in the post-anaesthesia or intensive care unit.

## 9. Risks of Active Warming Therapy

Perioperative warming therapy is generally very safe, and the advantages massively outweigh the rare adverse events that occur with active warming therapy. The most common adverse event associated with active warming is inefficient warming, which contributes to perioperative hypothermia despite active warming therapy. In most cases, this problem can be overcome by a more adequate warming method.

### 9.1. Risks of Forced-Air Warming

#### 9.1.1. Thermal Softening of Tracheal Tubes

During forced-air warming, the softening and kinking of tracheal tubes may occur, and increases in airway pressures up to complete obstruction of the airway have been observed [95]. This can cause ventilatory problems if not recognized immediately.

#### 9.1.2. Burns

**Figure 6:**
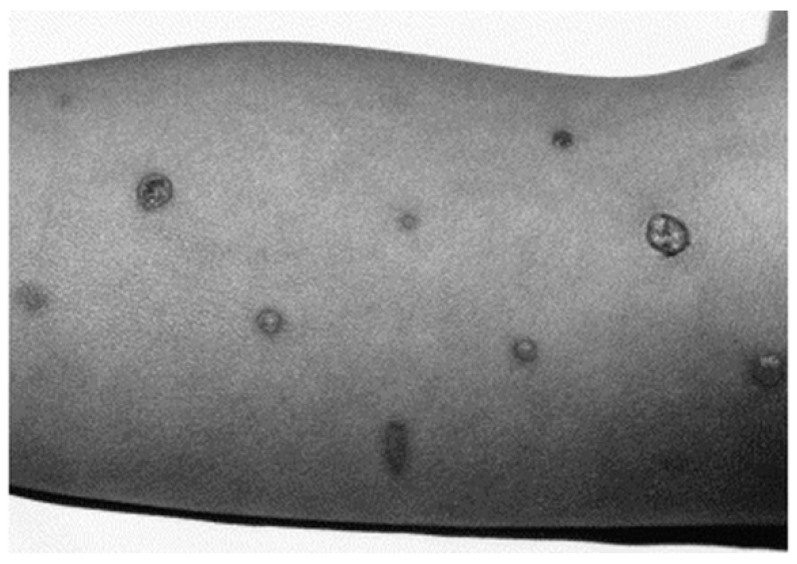
Second-degree burn of a 3-year-old boy after complex cardiac surgery with critically reduced peripheral perfusion. Reproduced from Truell KD, Bakerman PR, Teodori MF, Maze A. Third-degree burns to intraoperative use of a Bair Hugger warming device. Ann Thorac Surg 200; 69: 1933–1934 with permission of Elsevier [100].

Burns are the most feared complications of active warming therapy. Correct forced-air warming usage is a remarkably safe procedure with about 15 to 25 million uses per year and only single cases of burns reported per year [96]. The most common cause of burns reported with the use of forced-air warmers is the use of a power unit without an adequate blanket [97]. This unintended use of a power unit is clearly a device misuse and is prohibited. Using the device as intended dramatically reduces the risk of burns. However, instances of burns have been published even with a blanket connected to the power unit. These burns may be caused by direct contact of the nozzle with the skin [98] as the nozzle is one of the warmest parts of the device. It may also occur when peripheral tissue perfusion is critically reduced (Figure 6) [99,100,101].

### 9.2. Noise

There are complaints about noise created by forced-air warmers that may disturb the concentration of the surgical team [102]. These devices can produce noise up to 84 dB [103]. However, in most hospitals, this is not a big problem. Electrical conductive heating mattresses produce less noise, but have not been evaluated in small children.

### 9.3. Risks of Conductive Warming

**Figure 7:**
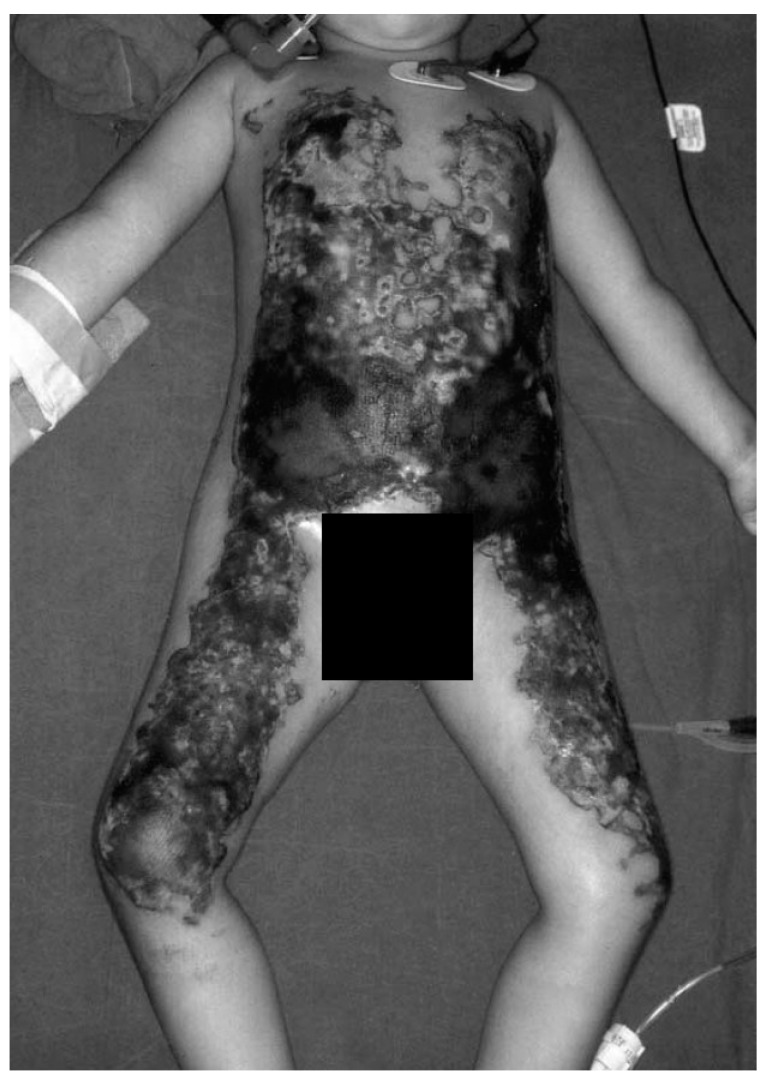
Third-degree burn of a 3-year-old girl after a neurosurgical procedure. As a result of a malfunctioning device, a large full-skin burn occurred. Reprinted from Acikel C, Kale B, Celikoz B. Major thermal burn due to intraoperative heating blanket malfunction. Burns 2002; 28: 283–284 with permission of Elsevier [106].

Conductive warming also carries a risk of burns. Especially the combination of pressure and heat can increase the risk of burns. Early reports of burns with conductive warming date from the 1960s [104] and several horrible cases have been described involving children receiving third-degree burns (Figure 7) [105,106].

### 9.4. Risks of Infusion Warming

#### 9.4.1. Risk of Burns

If the infused fluids are too hot, then infusion warming can also lead to full-thickness burns and venous thrombosis [107]. Additionally, close contact of the infusion warmer line with the skin can result in burns [108].

#### 9.4.2. Risks of Infections and Haemolysis

Infusion warming devices that use water as a heat exchanging medium may carry a risk of infectious complications. In water from counter-current warmers, more than 100,000 colonies of Gram-negative organisms have been found that may cause infections [109]. When leaks within counter-current infusion tubing occur, there can be a mixing of the sterile infusion and the bacterially contaminated warming fluid [110,111]. This may lead to bacteriaemia, electrolyte disturbances, and haemolysis [111].

#### 9.4.3. Air Embolism

**Figure 8:**
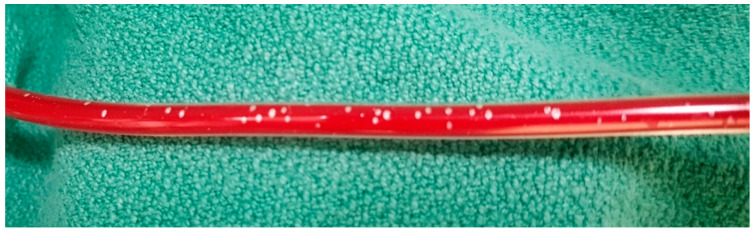
Gas bubbles in a heated fluid.

A general problem of infusion warming is that, with increasing temperature, the solubility of gases in fluids or blood decreases, potentially leading to nitrogen-containing air bubbles. Additionally, if an infusion fluid contains small bubbles, these bubbles will expand when the fluid is heated [109]. These gas bubbles can frequently be seen with the use of infusion warmers (Figure 8).

These air bubbles can be trapped in the pulmonary circulation and platelets may aggregate at the air–blood interface of pulmonary arterial vessels, causing microthrombi. Normally these small air emboli are not associated with clinical problems in children. However, children with congenital heart diseases that allow right to left shunting (e.g., patent foramen ovale, atrial and ventricular septal defects, transposition of the great arteries, tetralogy of Fallot, etc.) have a risk of air embolism to the brain or other organs. On the other hand, it must be stressed that outgassing is a process that occurs with the warming of cold fluids, and happens inside the body when the fluid is infused without warming.

### 9.5. Overheating

Especially during longer-lasting operations in small children, forced-air warming can lead to overheating [17,81,82,88,112]. Intraoperative hyperthermia has been associated with SSI in neonates and infants [57] and is generally undesirable. Therefore, it is imperative to monitor core temperature in children. If the core body temperature in a normothermic child rises steadily, then the power unit must be adjusted. As a rule of thumb, the temperature of the power unit can be reduced to 32–34 °C when the core temperature reaches 37 °C.

## 10. Conclusions

Although paediatric anaesthesiologists were the first to describe the adverse effects of perioperative hypothermia, and the first to implement effective measures against perioperative hypothermia, children today still often become hypothermic. This is due to their vulnerable physiology, but also due to insufficient perioperative warming.

A good perioperative warming strategy must be planned in advance including maintenance of normothermia during transportation, active warming before induction of anaesthesia, active warming during anaesthesia and surgery, and accurate measurement of core temperature. Infusion warming is only needed when larger amounts of fluids must be administered. Core temperature measurements must be used to control the efficacy of active warming therapy and to prevent overheating of children.

## Figures and Tables

**Table 1:** Formulas to estimate oesophageal temperature probe insertion depth in children.

Authors	Based on	Formula
Whitby et al. [64]	Age (years)	2 × age (years)/3 + 10 [cm] from corniculate cartilages
Hong et al. [65]	Height (cm)	(a) Best fit formula: 0.180 × height + 6.749 (cm) (b) Simplified formula: height/5 + 5 (cm) from the incisors, respectively

**Table 2:** Recommendations on core temperature monitoring.

Pre-OP	Intra-OP (General Anaesthesia)	Post-OP
Oral, axillary (rectal) (Tympanic)	Continuous methods: Oesophageal Nasopharyngeal Non-invasive methods based on ZHF technology Bladder, rectal	Serial measurements: Oral, axillary (rectal) (Tympanic) Hypothermic patients: Continuous methods, e.g., non-invasive methods based on ZHF technology Intubated patients: see Intra-OP

## Data Availability

Not applicable.

## References

[1] Torossian A., Becke K., Bein B., Bräuer A., Gantert D., Greif R., Höcker J., Horn E.P., Kimberger O., Klar E. S3 Leitlinie “Vermeidung von perioperativer Hypothermie”: Aktualisierung 2019. https://www.awmf.org/uploads/tx_szleitlinien/001-018l_S3_Vermeidung_perioperativer_Hypothermie_2019-08.pdf.

[2] von Bibra E.F., Harless E. (1847). Die Wirkung des Schwefeläthers in Chemischer und Physiologischer Beziehung.

[3] France G.G. (1957). Hypothermia in the newborn: Body temperatures following anaesthesia. Br. J. Anaesth..

[4] Farman J.V. (1962). Heat losses in infants undergoing surgery in air-conditioned theatres. Br. J. Anaesth..

[5] Bering E.A., Matson D.D. (1953). A technic for the prevention of severe hypothermia during surgery of infants. Ann. Surg..

[6] Calvert D.G. (1962). Inadvertent hypothermia in paediatric surgery and a method for its prevention. Anaesthesia.

[7] Lewis R.B., Shaw A., Etchells A.H. (1973). Contact mattress to prevent heat loss in neonatal and paediatric surgery. Br. J. Anaesth..

[8] Görges M., Afshar K., West N., Pi S., Bedford J., Whyte S.D. (2019). Integrating intraoperative physiology data into outcome analysis for the ACS Pediatric National Surgical Quality Improvement Program. Paediatr. Anaesth..

[9] Pearce B., Christensen R., Voepel-Lewis T. (2010). Perioperative Hypothermia in the Pediatric Population: Prevalence, Risk Factors and Outcomes. J. Anesth. Clin. Res..

[10] Sim R., Hall N.J., de Coppi P., Eaton S., Pierro A. (2012). Core temperature falls during laparotomy in infants with necrotizing enterocolitis. Eur. J. Pediatr. Surg..

[11] Cui Y., Wang Y., Cao R., Li G., Deng L., Li J. (2020). The low fresh gas flow anesthesia and hypothermia in neonates undergoing digestive surgeries: A retrospective before-after study. BMC Anesthesiol..

[12] Ongun E.A., Dursun O., Kazan M.S., Nur B., Mihci E. (2018). Early postoperative follow-up after craniosynostosis surgery. Turk. J. Med. Sci..

[13] Thompson D.R., Zurakowski D., Haberkern C.M., Stricker P.A., Meier P.M., Bannister C., Benzon H., Binstock W., Bosenberg A., Brzenski A. (2018). Endoscopic Versus Open Repair for Craniosynostosis in Infants Using Propensity Score Matching to Compare Outcomes: A Multicenter Study from the Pediatric Craniofacial Collaborative Group. Anesth. Analg..

[14] Brozanski B.S., Piazza A.J., Chuo J., Natarajan G., Grover T.R., Smith J.R., Mingrone T., McClead R.E., Rakesh R., Rintoul N. (2020). STEPP IN: Working Together to Keep Infants Warm in the Perioperative Period. Pediatrics.

[15] Schroeck H., Lyden A.K., Benedict W.L., Ramachandran S.K. (2016). Time Trends and Predictors of Abnormal Postoperative Body Temperature in Infants Transported to the Intensive Care Unit. Anesthesiol. Res. Prac..

[16] Kim P., Taghon T., Fetzer M., Tobias J.D. (2013). Perioperative hypothermia in the pediatric population: A quality improvement project. Am. J. Med. Qual..

[17] Nemeth M., Lovric M., Asendorf T., Bräuer A., Miller C. (2020). Intraoperative zero-heat-flux thermometry overestimates esophageal temperature by 0.26 °C: An observational study in 100 infants and young children. J. Clin. Monit. Comput..

[18] Hubbard R., Edmonds K., Rydalch E., Pawelek O., Griffin E., Gautam N. (2020). Anesthetic management of catheter-based patent ductus arteriosus closure in neonates weighing <3 kg: A Retrospective Ob-servational Study. Paediatr. Anaesth..

[19] Galante D. (2007). Intraoperative hypothermia. Relation between general and regional anesthesia, upper- and lower-body warming: What strategies in pediatric anesthesia?. Paediatr. Anaesth..

[20] Sessler D.I. (2008). Temperature monitoring and perioperative thermoregulation. Anesthesiology.

[21] Sessler D.I. (2016). Perioperative thermoregulation and heat balance. Lancet.

[22] Knobel R., Holditch-Davis D. (2007). Thermoregulation and heat loss prevention after birth and during neonatal intensive-care unit stabilization of extremely low-birthweight infants. J. Obstet. Gynecol. Neonatal Nurs..

[23] Wankhade U.D., Shen M., Yadav H., Thakali K.M. (2016). Novel Browning Agents, Mechanisms, and Therapeutic Potentials of Brown Adipose Tissue. BioMed Res. Int..

[24] Cypess A.M., Lehman S., Williams G., Tal I., Rodman D., Goldfine A.B., Kuo F.C., Palmer E.L., Tseng Y.-H., Doria A. (2009). Identification and importance of brown adipose tissue in adult humans. N. Engl. J. Med..

[25] Lapa C., Maya Y., Wagner M., Arias-Loza P., Werner R.A., Herrmann K., Higuchi T. (2015). Activation of brown adipose tissue in hypothyroidism. Ann. Med..

[26] Dawkins M.J., Scopes J.W. (1965). Non-shivering thermogenesis and brown adipose tissue in the human new-born infant. Nature.

[27] Houstĕk J., Vízek K., Pavelka S., Kopecký J., Krejcová E., Hermanská J., Cermáková M. (1993). Type II iodothyronine 5′-deiodinase and uncoupling protein in brown adipose tissue of human newborns. J. Clin. Endocrinol. Metab..

[28] Lyon A.J., Pikaar M.E., Badger P., McIntosh N. (1997). Temperature control in very low birthweight infants during first five days of life. ADC Fetal Neonatal Ed..

[29] Bissonnette B., Sessler D.I. (1992). Thermoregulatory thresholds for vasoconstriction in pediatric patients anesthetized with halothane or halothane and caudal bupivacaine. Anesthesiology.

[30] Plattner O., Semsroth M., Sessler D.I., Papousek A., Klasen C., Wagner O. (1997). Lack of nonshivering thermogenesis in infants anesthetized with fentanyl and propofol. Anesthesiology.

[31] Sessler D.I. (2013). Forced-air warming in infants and children. Paediatr. Anaesth..

[32] Sedin G., Okken A., Koch J. (1995). Neonatal Heat Transfer, Routes of Heat Loss and Heat Gain. Thermoregulation of Sick and Low Birth Weight Neonates: Temperature Control Temperature Monitoring Thermal Environment.

[33] Matsukawa T., Hanagata K., Ozaki M., Iwashita H., Koshimizu M., Kumazawa T.I.M. (1997). midazolam as premedication produces a concentration-dependent decrease in core temperature in male volunteers. Br. J. Anaesth..

[34] Sessler D.I. (2000). Perioperative heat balance. Anesthesiology.

[35] Bräuer A. (2017). Perioperative Temperature Management.

[36] Lai L.-L., See M.-H., Rampal S., Ng K.-S., Chan L. (2019). Significant factors influencing inadvertent hypothermia in pediatric anesthesia. J. Clin. Monit. Comput..

[37] Dicker A., Ohlson K.B., Johnson L., Cannon B., Lindahl S.G., Nedergaard J. (1995). Halothane selectively inhibits nonshivering thermogenesis. Possible implications for thermoregulation during anesthesia of infants. Anesthesiology.

[38] Tander B., Baris S., Karakaya D., Ariturk E., Rizalar R., Bernay F. (2005). Risk factors influencing inadvertent hypothermia in infants and neonates during anesthesia. Paediatr. Anaesth..

[39] Morehouse D., Williams L., Lloyd C., McCoy D.S., Miller Walters E., Guzzetta C.E., Baumgart S., Sill A., Mueller-Burke D., Short B.L. (2014). Perioperative hypothermia in NICU infants: Its occurrence and impact on infant outcomes. Adv. Neonatal. Care.

[40] World Health Organization (1997). Maternal; Newborn Health/Safe Motherhood. Thermal Protection of the Newborn: A Practical Guide.

[41] Engorn B.M., Kahntroff S.L., Frank K.M., Singh S., Harvey H.A., Barkulis C.T., Barnett A.M., Olambiwonnu O.O., Heitmiller E.S., Greenberg R.S. (2017). Perioperative hypothermia in neonatal intensive care unit patients: Effectiveness of a thermoregulation intervention and associated risk factors. Paediatr. Anaesth..

[42] Feltes T.F., Bacha E., Beekman R.H., Cheatham J.P., Feinstein J.A., Gomes A.S., Hijazi Z.M., Ing F.F., de Moor M., Morrow W.R. (2011). Indications for cardiac catheterization and intervention in pediatric cardiac disease: A scientific statement from the American Heart Association. Circulation.

[43] Sun Z., Honar H., Sessler D.I., Dalton J.E., Yang D., Panjasawatwong K., Deroee A.F., Salmasi V., Saager L., Kurz A. (2015). Intraoperative core temperature patterns, transfusion requirement, and hospital duration in patients warmed with forced air. Anesthesiology.

[44] Mullany L.C., Katz J., Khatry S.K., LeClerq S.C., Darmstadt G.L., Tielsch J.M. (2010). Risk of mortality associated with neonatal hypothermia in southern Nepal. Arch Pediatr. Adolesc. Med..

[45] Brindle M.E., McDiarmid C., Short K., Miller K., MacRobie A., Lam J.Y.K., Brockel M., Raval M.V., Howlett A., Lee K.-S. (2020). Consensus Guidelines for Perioperative Care in Neonatal Intestinal Surgery: Enhanced Recovery After Surgery (ERAS®) Society Recommendations. World J. Surg..

[46] Sessler D.I. (2001). Complications and treatment of mild hypothermia. Anesthesiology.

[47] Kurz A., Sessler D.I., Lenhardt R. (1996). Perioperative normothermia to reduce the incidence of surgical-wound infection and shorten hospitalization. Study of Wound Infection and Temperature Group. N. Engl. J. Med..

[48] Madar J., Roehr C.C., Ainsworth S., Ersdal H., Morley C., Rüdiger M., Skåre C., Szczapa T., Te Pas A., Trevisanuto D. (2021). European Resuscitation Council Guidelines 2021: Newborn resuscitation and support of transition of infants at birth. Resuscitation.

[49] Moler F.W., Silverstein F.S., Holubkov R., Slomine B.S., Christensen J.R., Nadkarni V.M., Meert K.L., Clark A.E., Browning B., Pemberton V.L. (2015). Therapeutic hypothermia after out-of-hospital cardiac arrest in children. N. Engl. J. Med..

[50] Moler F.W., Silverstein F.S., Holubkov R., Slomine B.S., Christensen J.R., Nadkarni V.M., Meert K.L., Browning B., Pemberton V.L., Page K. (2017). Therapeutic Hypothermia after In-Hospital Cardiac Arrest in Children. N. Engl. J. Med..

[51] Field D., Juszczak E., Linsell L., Azzopardi D., Cowan F., Marlow N., Edwards D. (2013). Neonatal ECMO study of temperature (NEST): A randomized controlled trial. Pediatrics.

[52] Hutchison J.S., Ward R.E., Lacroix J., Hébert P.C., Barnes M.A., Bohn D.J., Dirks P.B., Doucette S., Fergusson D., Gottesman R. (2008). Hypothermia therapy after traumatic brain injury in children. N. Engl. J. Med..

[53] Deshpande S.A., Platt M.P. (1997). Association between blood lactate and acid-base status and mortality in ventilated babies. ADC Fetal Neonatal Ed..

[54] Miller S.S., Lee H.C., Gould J.B. (2011). Hypothermia in very low birth weight infants: Distribution, risk factors and outcomes. J. Perinatol..

[55] Görges M., West N.C., Cheung W., Zhou G., Miyanji F., Whyte S.D. (2016). Preoperative warming and undesired surgical and anesthesia outcomes in pediatric spinal surgery—A retrospective cohort study. Paediatr. Anaesth..

[56] Linam W.M., Margolis P.A., Staat M.A., Britto M.T., Hornung R., Cassedy A., Connelly B.L. (2009). Risk factors associated with surgical site infection after pediatric posterior spinal fusion procedure. Infect. Control Hosp. Epidemiol..

[57] Walker S., Amin R., Arca M.J., Datta A. (2020). Effects of intraoperative temperatures on postoperative infections in infants and neonates. J. Pediatr. Surg..

[58] Sessler D.I. (2021). Perioperative Temperature Monitoring. Anesthesiology.

[59] Rubinstein E.H., Sessler D.I. (1990). Skin-surface temperature gradients correlate with fingertip blood flow in humans. Anesthesiology.

[60] Burgess G.E., Cooper J.R., Marino R.J., Peuler M.J. (1978). Continuous monitoring of skin temperature using a liquid-crystal thermometer during anesthesia. South. Med. J..

[61] Bloch E.C., Ginsberg B., Binner R.A. (1993). The esophageal temperature gradient in anesthetized children. J. Clin. Monit..

[62] Wang M., Singh A., Qureshi H., Leone A., Mascha E.J., Sessler D.I. (2016). Optimal Depth for Nasopharyngeal Temperature Probe Positioning. Anesth. Analg..

[63] Pasquier M., Paal P., Kosinski S., Brown D., Podsiadlo P., Darocha T. (2020). Esophageal Temperature Measurement. N. Engl. J. Med..

[64] Whitby J.D., Dunkin L.J. (1970). Oesophageal temperature differences in children. Br. J. Anaesth..

[65] Hong S.H., Lee J., Jung J.-Y., Shim J.W., Jung H.S. (2020). Simple calculation of the optimal insertion depth of esophageal temperature probes in children. J. Clin. Monit. Comput..

[66] Roth J.V., Braitman L.E. (2008). Nasal temperature can be used as a reliable surrogate measure of core temperature. J. Clin. Monit. Comput..

[67] Lee J., Lim H., Son K.-G., Ko S. (2014). Optimal nasopharyngeal temperature probe placement. Anesth. Analg..

[68] Snoek A.P., Saffer E. (2016). Agreement between lower esophageal and nasopharyngeal temperatures in children ventilated with an endotracheal tube with leak. Pediatr. Anesth..

[69] Imamura M., Matsukawa T., Ozaki M., Sessler D.I., Nishiyama T., Kumazawa T. (1998). The accuracy and precision of four infrared aural canal thermometers during cardiac surgery. Acta Anaesthesiol. Scand..

[70] Langham G.E., Maheshwari A., Contrera K., You J., Mascha E., Sessler D.I. (2009). Noninvasive temperature monitoring in postanesthesia care units. Anesthesiology.

[71] Greenes D.S., Fleisher G.R. (2001). Accuracy of a noninvasive temporal artery thermometer for use in infants. Arch Pediatr. Adolesc. Med..

[72] Carvalho H., Najafi N., Poelaert J. (2019). Intra-operative temperature monitoring with cutaneous zero-heat- flux-thermometry in comparison with oesophageal temperature: A prospective study in the paediatric population. Paediatr. Anaesth..

[73] Evron S., Weissman A., Toivis V., Shahaf D.B., You J., Sessler D.I., Ezri T. (2017). Evaluation of the Temple Touch Pro, a Novel Noninvasive Core-Temperature Monitoring System. Anesth. Analg..

[74] Conway A., Bittner M., Phan D., Chang K., Kamboj N., Tipton E., Parotto M. (2020). Accuracy and precision of zero-heat-flux temperature measurements with the 3M™ Bair Hugger™ Temperature Monitoring System: A systematic review and meta-analysis. J. Clin. Monit. Comput..

[75] Cassey J.G., King R.A., Armstrong P. (2010). Is there thermal benefit from preoperative warming in children?. Paediatr. Anaesth..

[76] Bräuer A., Perl T., Uyanik Z., English M.J.M., Weyland W., Braun U. (2004). Perioperative thermal insulation: Minimal clinically important differences?. Br. J. Anaesth..

[77] NICE Hypothermia: Prevention and Management in Adults Having Surgery: Clinical Guideline. www.nice.org.uk/guidance/cg65.

[78] Matsukawa T., Sessler D.I., Sessler A.M., Schroeder M., Ozaki M., Kurz A., Cheng C. (1995). Heat flow and distribution during induction of general anesthesia. Anesthesiology.

[79] Horn E.-P., Bein B., Böhm R., Steinfath M., Sahili N., Höcker J. (2012). The effect of short time periods of pre-operative warming in the prevention of peri-operative hypothermia. Anaesthesia.

[80] Eich C., Zink W., Schwarz S., Radke O.C., Bräuer A. (2009). A combination of convective and conductive warming ensures pre- and post-bypass normothermia in paediatric cardiac anaesthesia. Appl. Cardiopulm. Pathophysiol..

[81] Triffterer L., Marhofer P., Sulyok I., Keplinger M., Mair S., Steinberger M., Klug W., Kimberger O. (2016). Forced-Air Warming During Pediatric Surgery: A Randomized Comparison of a Compressible with a Noncompressible Warming System. Anesth. Analg..

[82] Witt L., Dennhardt N., Eich C., Mader T., Fischer T., Bräuer A., Sümpelmann R. (2013). Prevention of intraoperative hypothermia in neonates and infants: Results of a prospective multicenter observational study with a new forced-air warming system with increased warm air flow. Paediatr. Anaesth..

[83] Kurz A., Kurz M., Poeschl G., Faryniak B., Redl G., Hackl W. (1993). Forced-air warming maintains intraoperative normothermia better than circulating-water mattresses. Anesth. Analg..

[84] Grote R., Wetz A., Bräuer A., Menzel M. (2020). Short interruptions between pre-warming and intraoperative warming are associated with low intraoperative hypothermia rates. Acta Anaesthesiol. Scand..

[85] Bräuer A., Scheithauer S. (2016). Prävention der unbeabsichtigten perioperativen Hypothermie. Krankenh.hyg. Up2Date.

[86] Bissonnette B., Sessler D.I., LaFlamme P. (1989). Passive and active inspired gas humidification in infants and children. Anesthesiology.

[87] Sümpelmann R., Becke K., Zander R., Witt L. (2019). Perioperative fluid management in children: Can we sum it all up now?. Curr. Opin. Anaesthesiol..

[88] Fillies T., Homann C., Meyer U., Reich A., Joos U., Werkmeister R. (2007). Perioperative complications in infant cleft repair. Head Face Med..

[89] Shorrab A.A., El-Sawy M.E., Othman M.M., Hammouda G.E. (2007). Prevention of hypothermia in children under combined epidural and general anesthesia: A comparison between upper- and lower-body warming. Paediatr. Anaesth..

[90] Beebe D.S., Beck D., Belani K.G. (1994). Clinical management of infants and newborn babies undergoing major surgery utilizing a rapid infusion device. Pediatr. Anesth..

[91] Bissonnette B., Paut O. (2002). Active warming of saline or blood is ineffective when standard infusion tubing is used: An experimental study. Can. J. Anesth..

[92] Schnoor J., Weber I., Macko S., Heussen N., Rossaint R. (2006). Heating capabilities of the Hotline and Autoline at low flow rates. Paediatr. Anaesth..

[93] Schmidt J.H., Weyland W., Fritz U., Bräuer A., Rathgeber J., Braun U. (1996). Experimentelle Untersuchung zur Effektivität verschiedener Infusions- und Blutwärme-verfahren. Anaesthesist.

[94] Perl T., Kunze-Szikszay N., Bräuer A., Quintel M., Röhrig A.L., Kerpen K., Telgheder U. (2019). Aluminium release by coated and uncoated fluid-warming devices. Anaesthesia.

[95] Ayala J.L., Coe A. (1997). Thermal softening of tracheal tubes: An unrecognized hazard of the Bair Hugger active patient warming system. Br. J. Anaesth..

[96] Bräuer A., Quintel M. (2009). Forced-air warming: Technology, physical background and practical aspects. Curr. Opin. Anaesthesiol..

[97] Mehta S.P. (2013). Burn injuries from warming devices in the operating room. ASA Monit..

[98] Azzam F.J., Krock J.L. (1995). Thermal burns in two infants associated with a forced air warming system. Anesth. Analg..

[99] Siddik-Sayyid S.M., Abdallah F.W., Dahrouj G.B. (2008). Thermal burns in three neonates associated with intraoperative use of Bair Hugger warming devices. Paediatr. Anaesth..

[100] Truell K.D., Bakerman P.R., Teodori M.F., Maze A. (2000). Third-degree burns due to intraoperative use of a Bair Hugger warming device. Ann. Thorac. Surg..

[101] Golden S., Bachmann C. (2006). Forced air warmer burn can occur with poor circulation. APSF Newsl..

[102] Wagner K., Swanson E., Raymond C.J., Smith C.E. (2008). Comparison of two convective warming systems during major abdominal and orthopedic surgery. Can. J. Anesth..

[103] Katz J.D. (2014). Noise in the operating room. Anesthesiology.

[104] Crino M.H., Nagel E.L. (1968). Thermal burns caused by warming blankets in the operating room. Anesthesiology.

[105] Dewar D.J., Fraser J.F., Choo K.L., Kimble R.M. (2004). Thermal injuries in three children caused by an electrical warming mattress. Br. J. Anaesth..

[106] Acikel C., Kale B., Celikoz B. (2002). Major thermal burn due to intraoperative heating blanket malfunction. Burns.

[107] Sieunarine K., White G.H. (1996). Full-thickness burn and venous thrombosis following intravenous infusion of microwave-heated crystalloid fluids. Burns.

[108] Arrandale L., Ng L. (2009). Superficial burn caused by a Hotline fluid warmer infusion set. Anaesthesia.

[109] (1996). In-line blood/solution warmers. Health Devices.

[110] Clarke P.A., Thornton M.J. (2009). Failure of a water-bath design intravenous fluid warmer. Can. J. Anaesth..

[111] Wilson S., Szerb J. (2007). Failure of an iv fluid warming device. Can. J. Anaesth..

[112] Mittnacht A.J.C., Lin H.-M., Liu X., Wax D. (2020). New-onset intra-operative hyperthermia in a large surgical patient population: A retrospective observational study. Eur. J. Anaesthesiol..

